# Physiological plateaus during normal labor and birth: A novel definition

**DOI:** 10.1111/birt.12843

**Published:** 2024-05-27

**Authors:** Marina Weckend, Kylie McCullough, Christine Duffield, Sara Bayes, Clare Davison

**Affiliations:** ^1^ School of Nursing and Midwifery Edith Cowan University Joondalup Western Australia Australia; ^2^ Faculty of Health University of Technology Sydney Ultimo New South Wales Australia; ^3^ Faculty of Health Sciences, School of Nursing, Midwifery and Paramedicine Australian Catholic University Fitzroy Victoria Australia

**Keywords:** failure to progress, labor dystocia, labor progress, midwifery, natural childbirth, normal birth, uterine inertia

## Abstract

**Background:**

Diagnoses of labor dystocia, and subsequent labor augmentation, make one of the biggest contributions to childbirth medicalization, which remains a key challenge in contemporary maternity care. However, labor dystocia is poorly defined, and the antithetical concept of physiological plateaus remains insufficiently explored.

**Aim:**

To generate a definition of physiological plateaus as a basis for further research.

**Methods:**

This qualitative study applied grounded theory methods and comprised interviews with 20 midwives across Australia, conducted between September 2020 and February 2022. Data were coded in a three‐phase approach, starting with inductive line‐by‐line coding, which generated themes and subthemes, and finally, through axial coding.

**Results:**

Physiological plateaus represent a temporary slowing of one or multiple labor processes and appear to be common during childbirth. They are reported throughout the entire continuum of labor, typically lasting between a few minutes to several hours. Their etiology/function appears to be a self‐regulatory mechanism of the mother‐infant dyad. Physiological plateaus typically self‐resolve and are followed by a self‐resumption of labor. Women with physiological plateaus during labor appear to experience positive birth outcomes.

**Discussion:**

Despite appearing to be common, physiological plateaus are insufficiently recognized in contemporary childbirth discourse. Consequently, there seems to be a significant risk of misinterpretation of physiological plateaus as labor dystocia. While findings are limited by the qualitative design and require validation through further quantitative research, the proposed novel definition provides an important starting point for further investigation.

**Conclusion:**

A better understanding of physiological plateaus holds the potential for a de‐medicalization of childbirth through preventing unjustified labor augmentation.

## Graphical abstract

Physiological plateaus appear to be common in labor, but remain insufficiently recognized in contemporary birth culture. Such plateaus are at risk of being misinterpreted as labour dystocia, leading to unjustified labour augmentation and caesarean sections. A new definition of plateaus seeks to provide clarity on this phenomenon, and promote further research.

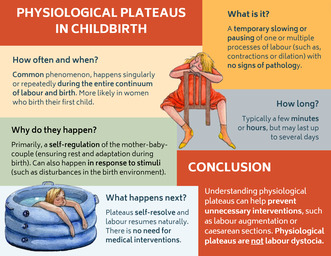

## 1 INTRODUCTION

One of the most pressing concerns in contemporary maternity care globally is the overuse, and frequently unnecessary use, of medical interventions during childbirth.^1^, ^2^ Commonly referred to as childbirth medicalization, this affects birthing women in high‐resource and low‐resource countries alike.^1^, ^3^ Childbirth medicalization is reflected in high rates of cesarean sections, where problems arise from associated intervention cascades and preventable poor birth outcomes for women and newborns.^4^

One of the biggest contributions to high cesarean section rates is made by the diagnosis of labor dystocia (also “*failure to progress”*).^5^, ^6^, ^7^, ^8^ This diagnosis commonly results in medical interventions, such as continuous cardiotocography, repeat vaginal examinations, augmentation of labor with oxytocin, and artificial rupture of membranes. Consequently, women who are diagnosed with labor dystocia are more likely to experience an instrumental birth (ventouse/forceps) or cesarean section.^9^, ^10^, ^11^

However, to date, labor dystocia has not been universally defined.^12^, ^13^ The “foundation of a dystocia diagnosis” remains the “interpretation of [cervical] dilation rates (cm/hour),” which represents a time‐based approach to assessing labor.^7^ ^(p.503)^ Various time‐based cut‐offs for cervical dilation are currently promoted, including 0.5 cm/hour, 1 cm/hour, or variable cm/hour rates depending on the phase of labor,^12^, ^14^, ^15^ despite limited evidence to support this. Consequently, diagnosing labor dystocia remains subjective and the use of oxytocin for labor augmentation has been described as “random” and “inexplicable.”^11^ ^(p.2),^ ^16^ ^(p.2)^ Some studies found that 42.5%–82.9% of women subjected to augmentation do not satisfy the indication criteria for this intervention^10^, ^11^, ^16^, ^17^— demonstrating that current maternity care approaches introduce preventable risk for a large proportion of women and infants.

Meanwhile, there is growing evidence that normal labor can be much slower than commonly assumed, can follow an inconsistent pattern with individual variety, and may involve times with slow progression.^18^, ^19^, ^20^ In light of this evidence, researchers and practitioners criticize the above‐described time‐based approach as reductionist and instead propose more fluid theories of labor processes and progression.^21^, ^22^, ^23^, ^24^, ^25^ Numerous variables can affect the pattern of contractions, the progress of cervical dilation, and fetal descent — rendering it rather unlikely that labor progresses linearly at a continuously increasing pace.^22^, ^25^, ^26^, ^27^ Instead, physiological labor may be more accurately represented through a model of alternating accelerating and decelerating phases, where each phase is a response to various internal and external stimuli. Such a multi‐factorial model acknowledges the existence of physiological plateaus, meaning periods of slowing and pausing labor patterns that constitute a physiological phenomenon of normal labor.

A recent literature review revealed significant conceptual and terminological variance surrounding the notion of physiological plateaus, and dozens of different terms/phrases and multiple concepts were found to co‐exist.^28^, ^29^ This lack of a definition contributes to a dearth of evidence surrounding physiological plateaus. Consequently, some guidelines for the management of slowing labor may not be evidence‐based. This lack of clarity must be addressed to build a foundation for further, much needed research in this area, which forms an important avenue for reducing unnecessary labor augmentation. This study aimed to define physiological plateaus, thereby providing researchers and practitioners with a starting point for exploring physiological plateaus in depth.

## 2 METHODS

### 2.1 Study design

The findings presented in this manuscript form one component of a larger qualitative research project, which followed a constructivist grounded theory methodology.^30^, ^31^ The overall aim of this research project was to investigate the properties and significance of physiological plateaus, which resulted in two main outputs: a sociological theory explaining maternity care providers' interactions surrounding physiological plateaus,^32^ and a novel definition of physiological plateaus (presented in this manuscript). The decision to report these two main outputs in separate manuscripts was made to ensure that underlying data can be reported with utmost transparency.

### 2.2 Participant eligibility and recruitment

Midwives in Australia with experience of supporting women during physiological childbirth were recruited through an advertisement, disseminated through the Australian College of Midwives and social media (Twitter, Facebook, LinkedIn) between August 2020 and June 2021. During screening, theoretical sampling was applied to ensure a diversity of perspectives, including a geographical spread and varying degrees of experience (years, work settings) of participants.

### 2.3 Data collection and analysis

Individual semi‐structured interviews were conducted between September 2020 and February 2022. Participants were encouraged to share case reports and their general views of physiological labor patterns/progress and physiological plateaus. During interviews, each participant's preferred terminology and conceptualization of physiological plateaus was used to prevent researcher bias. This included any concepts of slowing, stalling, or pausing labor that midwives interpreted as physiological.

All interviews were audio‐recorded with participants' consent, and manually transcribed. NVivo V.12 software was used for manual coding, applying constructivist grounded theory methods.^30^ First, transcripts were coded inductively line‐by‐line, fragmenting data into numerous individual codes. Then, similar codes were collapsed into themes and subthemes during focused coding. In a third step, data underwent deductive axial coding, collating information on the conceptual boundaries, prevalence, timing, duration, etiology/function, and consequences of physiological plateaus.

Trustworthiness of this study was ensured by applying researcher triangulation, data triangulation (constant comparative method), and internal quality audits. The coding and synthesis of findings were agreed by group consensus among all authors participating in the analysis.

## 3 RESULTS

### 3.1 Participants

Twenty midwives in New South Wales (*n* = 6), Victoria (*n* = 4), Queensland (*n* = 2), Western Australia (*n* = 7), and South Australia (*n* = 1) participated in this study. Participants had a median 14.5 years' practice experience (range: 2–40+ years) across various birth settings, including obstetric‐led and midwife‐led hospital units, birth centers, and homebirth services. At the time of data collection, 70% (*n* = 14) of participants were actively practicing as midwives, either independently (*n* = 5), in hospital (*n* = 5), in midwifery group practice (*n* = 2), in a birth center (*n* = 1), or in remote health services (*n* = 1). Of the remaining participants (30%, *n* = 6), three had left the midwifery profession to pursue other careers, two had retired and one worked as a midwifery lecturer.

Below, participants' views of physiological plateaus are presented according to axial coding categories (conceptual boundaries, prevalence, timing, duration, etiology/function, consequences), followed by a synthesis into a novel definition.

### 3.2 Conceptual boundaries of physiological plateaus

Participants conceptualized physiological plateaus as a temporary slowing or pausing of one or multiple labor processes, including uterine contractions, cervical dilation, maternal behavioral changes, and fetal positioning. Uterine contractions were described to decrease in frequency, duration, and/or intensity, as illustrated in the following quote:

More often than not, it's the frequency of the contractions … instead of having 30 … [to] 60 seconds to have a rest, it might give her four or five minutes. (Josh)

Further, midwives described a decreasing pace, complete halt, or reversal of cervical dilation during labor, whereby *reversal* referred to a temporary contraction of cervical tissue to a smaller overall diameter than previously examined. For example, Pauline, discussing the impact of stress hormones on labor physiology, shared this observation:

I've seen women who closed their cervix because they don't like their midwife … It wouldn't go from six centimeters to closed, but six to four maybe. (Pauline)

Participants also reported plateaus in what they understood as a natural sequence of maternal behavioral changes during labor. For example, a previously actively laboring woman may become tired and fall asleep during labor, as reported by this participant:

The contractions stopped for a while and she had a little sleep … and then she got up and walked again. (Quinn)

Finally, some midwives conceptualized a slowing fetal descent, or a temporary lack of change in the expected sequence of fetal positioning (e.g., flexion, rotation) as physiological plateaus. Key to the conceptualization of slowing and pausing labor as a physiological plateau (vs. a pathological process) was that both mother and fetus exhibited no signs of pathology in the lead‐up, during, and after the plateau. This included that the woman expresses no concern about her own labor pattern.

### 3.3 Estimated prevalence of physiological plateaus

Most participants emphasized that physiological plateaus were “super common” (Ellen, Noora), experienced by “most women” (Olivia, Isabelle) and observable “all the time” (Mary).

It's super common … It's not an abnormal thing, it's a normal thing that can occur. (Noora)

Nonetheless, participants reported variations in the prevalence of physiological plateaus, noting, for example, that plateaus appear to be more commonly observable in nulliparous women.

[Plateaus occur] probably more with first‐time mums … Maybe it happens with the second [birth], but it is not as obvious because … [the birth] is usually shorter (everything is condensed). (Ellen)

Further, the woman's personality and emotional state may affect whether plateaus occur, or how pronounced they are. Certain birth situations, such as a transfer from home to hospital, may also increase the prevalence of plateaus, as illustrated here:

[When transferring to hospital] 40 to 60% of women will need some time to become acclimatised before they motor on, so to speak. And I think a lot of that is all down to the personality of the person. People who are … confident … can relax and it's fine … And other people, who are … shy … just need to feel safe before they can continue. (Freya)

Participants mostly agreed that the prevalence of plateaus can vary depending on the unique combination of factors present during birth, including the baby's position, the woman's physical and psychological state, and even the woman's desire for a plateau.

### 3.4 Timing of physiological plateaus

Participants reported physiological plateaus during all phases of childbirth—from the onset of labor, through early and established labor (first stage), transition and birth of the baby (second stage), until the birth of the placenta (third stage).

During early labor, participants interpreted plateaus as a physiological phenomenon of fluent transitioning from the relative quiescence of pregnancy to the comparatively vigorous exercise of birthing. Participants described this early labor phase as a process that may take several days, or weeks. All midwives in this study interpreted plateaus during early labor as a normal occurrence that formed ‘accepted knowledge’ in contemporary maternity care culture.

Early labor would start‐stop … it's well accepted that there are pauses … in early labor (Mary)

During established/active labor midwives perceived physiological plateaus as a possible if not common phenomenon, albeit arguing that this understanding does not form accepted knowledge in the wider childbirth discourse, as explained by this participant:

There are pauses … that can happen in established labor. And it doesn't mean anything is wrong. It doesn't mean that the labor has suddenly become faulty … it's quite normal … But I guess, the pause [in established labor] … is not well understood. (Mary)

Participants also reported physiological plateaus during transition and second stage, associating these plateaus with processes of maternal psychological integration, fetal repositioning, and maternal energy gathering before the final pushing phase.

When you're hitting transition … quite often the body will say ‘Okay mum, have a breather now, collect your thoughts, collect your energy, because now the hard work starts.’ And that's not uncommon. (Josh)

Several midwives labeled these plateaus “rest and be thankful phase,” and all participants who reported these plateaus emphasized that midwives should facilitate rest and recovery during this time.

That rest and be thankful phase at the end, that's a very normal plateau … that all women … have. Maybe not all, but it's very … common. (Mary)

Further, several midwives reported that plateaus can occur during the period of bearing down and up to imminently before birth. In the absence of signs of pathology, this timing of plateaus was not considered abnormal or concerning, as exemplified by this reflection:

[When] a woman is fully dilated … regularly, I'll get … a good 5/10 minutes or so with just nothing. And then, maybe some slight contractions. And I always tell my mums that it's because her body is getting ready to have the last big part, that it's giving you a break. (Pauline)

Over half of participants explicitly reported physiological plateaus throughout all phases of labor and noted that a woman's labor may plateau several times without constituting pathology.

Your whole entire labor can stop and start and do whatever pattern it does right up until that point where you're almost imminently going to birth your baby. (Ryleigh)

And for some women it can happen several times throughout the one labor … it can happen at any stage. (Olivia)

Overall, participants' reports indicate that physiological plateaus may occur singularly or repeatedly at any time throughout the entire continuum of labor and birth.

### 3.5 Duration of physiological plateaus

Midwives in this study described a typical duration of physiological plateaus as being between a few minutes and several hours, although some plateaus reportedly last multiple days, as illustrated here:

Sometimes, they will just sleep for a few hours and then it all just picks up. Sometimes, it all just fizzles out and then there is another day or two before it starts up again. (Gabriella)

Participants understood physiological plateaus as a relaxing (not stressful) time for the woman and baby, arguing that the duration of plateaus was not a suitable indicator to assess whether the labor pattern was physiological or pathological. Instead, midwives based their assessment on fetal and maternal well‐being, as demonstrated here:

‘Is the mother safe and is the baby safe?’ … That's a really good framework to think from … if you can answer yes to both of those two things, time limits don't necessarily matter. (Ryleigh)

If … the contractions are what causes stress on mum and baby … [and] if that stress is not there, then, does it matter if we have a little bit of a pause before we go again? … It's not a stressful time for them; it's a relaxing time for them. (Isabelle)

Overall, midwives were satisfied that in the absence of signs of pathology, plateaus during labor are “a physiological process and … normal until proven otherwise” (Isabelle).

### 3.6 Etiology/function of physiological plateaus

**FIGURE 1:**
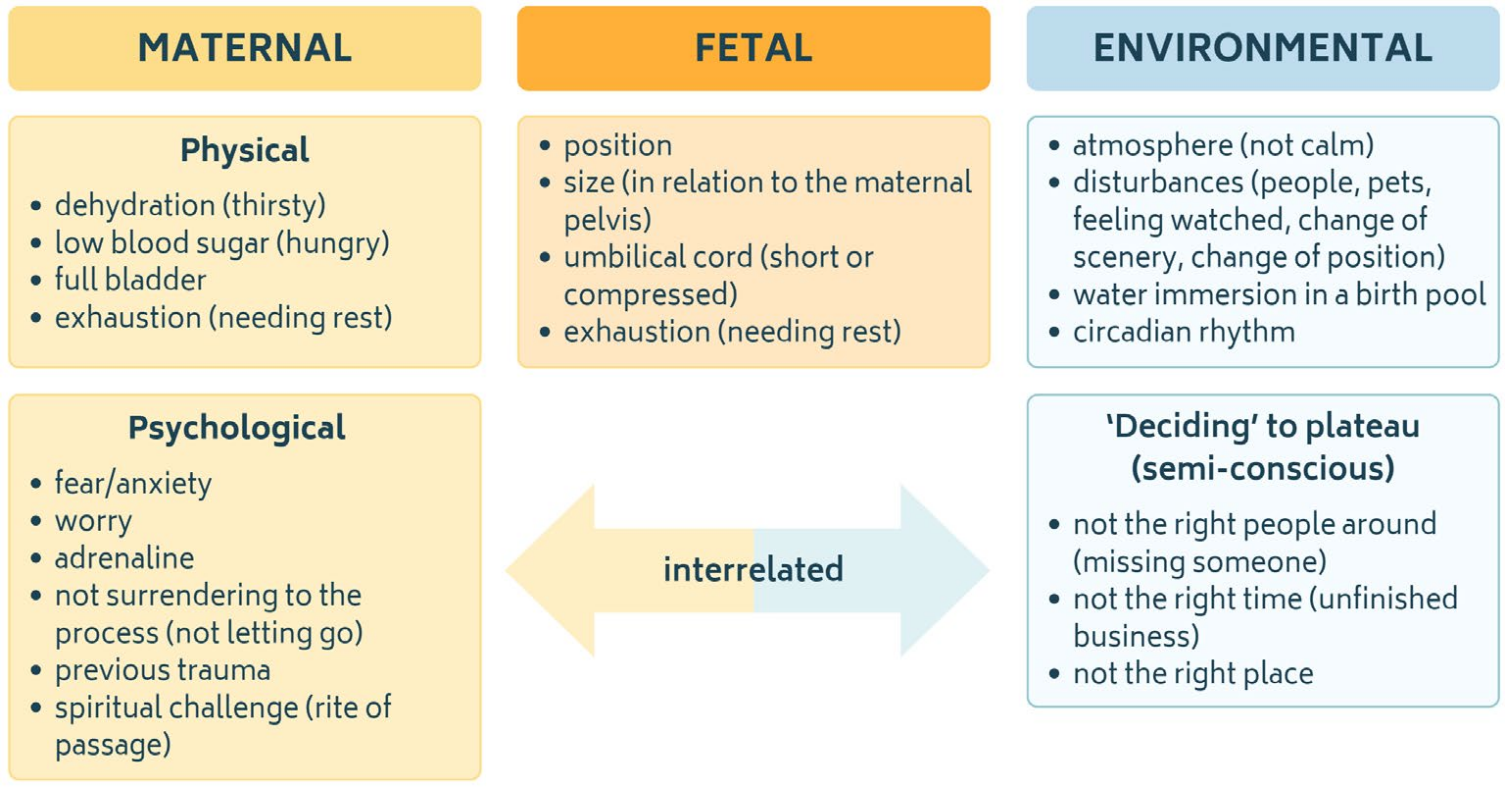
Factors reported by participants that may cause physiological plateaus during labor. [Colour figure can be viewed at wileyonlinelibrary.com]

Midwives in this study reported “lots of reasons” (Lina) why labor can plateau, including maternal, fetal, and environmental factors (Figure 1). Maternal physical factors included a low blood sugar, potential dehydration, a full bladder, or an overall need for rest, as shown in this example:

There's lots of reasons that labor can slow, stall or stop … [One is] decreased energy: If it's been a long labor, sometimes the body will go ‘Okay, need a rest, need some energy, pull back!’ (Lina)

Reported maternal psychological factors largely resolved around maternal anxiety or worry—emotions which midwives associated with an increase in adrenaline levels. Such feelings could reportedly persist throughout the course of labor or emerge intermittently, resulting in various slow or fluctuating labor patterns, or a sudden complete halt. Noora explains this as follows:

When women are scared, they pause. It's as simple as that. When women are anxious or worried or not sure what's happening, feel out of control, there can be periods of pauses. (Noora)

Maternal psychological factors were reported as being highly individual to the woman's personal history and life circumstances, as illustrated here:

Sometimes … it will be relationship stuff between the partner or the mother. Or … they've had postnatal depression last time and they're worried how they're gonna care for two babies under two. … Anything! (Amber)

Several participants entertained the idea whether women might be able to cause (and resolve) plateaus during labor somewhat consciously, explaining that women require ‘the right place, the right time and the right people’ to commence and sustain labor. Accordingly, if any of these parameters are “out of line,” this may cause labor to plateau (or may delay labor onset), as exemplified in this quote:

Your mind is so powerful that I have seen women stop labor. I have seen women delay, waiting for a certain person. (Gabriella)

Reported fetal factors that may cause plateaus comprised the fetal position, size, length of the umbilical cord, and a fetal need for recovery. Midwives acknowledged, for example, that unfavorable fetal positions may cause plateaus, as illustrated here:

[Her labor] stalled at about 7 to 8 cm, and the suspicion was that the baby was OP [occipito‐posterior] in that situation … You had the coupling of contractions and contractions had just died off (spaced out). (Ellen)

Further, environmental factors were quoted as typical causes of physiological plateaus, including the atmosphere of the birth environment, external disturbances, and the circadian rhythm. Reported disturbances included any factor that distracted the woman, for example, the presence of children in the birth space:

She was contracting and stopping and starting and was all over the shop. And it was just because she was looking after the toddler (Katrina)

Changes in scenery formed another typical disturbance. For example, midwives explained that when a woman travels to hospital, this involves changes of positions and possibly anxiety that may cause plateaus:

Typical! You know it's been hectic. The journey is obviously quite difficult for them, quite painful. And then, as soon as they're in that facility, that whole change of scene makes everything slow down. (Freya)

Finally, several participants emphasized that a causative factor of physiological plateaus may not always be identifiable. Such plateaus were often still interpreted as a naturally fluctuating labor pattern for individual women:

[For some] women and babies, it's just what their body does. They just get a break. And it's not for a reason in terms of a reason we need to fix … I just wait it out if mum is well and baby is well. Why not? (Isabelle)

In summary, numerous maternal, fetal, and environmental factors were reported as typical causes of physiological plateaus. Where in doubt or requiring a fresh perspective, participants reported conferring with experienced midwife colleagues to discuss individual labor patterns. Primarily, midwives conceptualized physiological plateaus as a mechanism of self‐regulation of the mother‐baby dyad, theorizing that physiological plateaus accommodate feto‐maternal rest, maternal psychological integration, and fetal repositioning. Secondarily, midwives understood some plateaus to be caused in response to internal and external disruptive stimuli, such as anxiety and disturbances in the birth environment.

### 3.7 Consequence of physiological plateaus

Participants explained that in 80%–90% of cases, physiological plateaus are followed by a natural resumption and acceleration of labor processes, and usually result in physiological birth outcomes for mother and baby, as exemplified here:

The labor started after about an hour and a half. Just started up again … she birthed this … good‐sized baby … and there was no problem at all. (Beatrice)

Say, if I'd cared for 300 women in ten years, maybe five [women had plateaus that did not self‐resolve]. Overwhelmingly, the majority, sort themselves out. (Amber)

Consequently, participants argued that physiological plateaus require no medical intervention. Many participants were concerned that contemporary maternity care guidelines may cause/exacerbate a risk of misinterpretation of physiological plateaus as labor dystocia, which then reportedly results in unnecessary medical interventions. One participant described her concern as follows:

At the end of the year, I sat down and worked out: 40% of my clients would have been offered some form of induction or augmentation based on hospital policies. None of them wanted that and they all went on to birth normally. (Ryleigh)

Consequently, many participants argued that physiological plateaus should be recognized as a normal element of labor, and women who experience plateaus should be assessed more holistically than what appears to be conventional in medicalized environments:

I think this [knowledge of plateaus] could really help women in obstetric models – if we could have evidence of ‘pauses are physiological.’ Cause we know it! Midwives who work with women know it. (Amber)

Overall, midwives in this study provided numerous case reports and explanations suggesting that physiological plateaus represent a transitory phenomenon during labor that typically self‐resolves, requires no medical interventions, and leads to positive birth outcomes for mother and child.

### 3.8 Novel definition of physiological plateaus in childbirth

**FIGURE 2:**
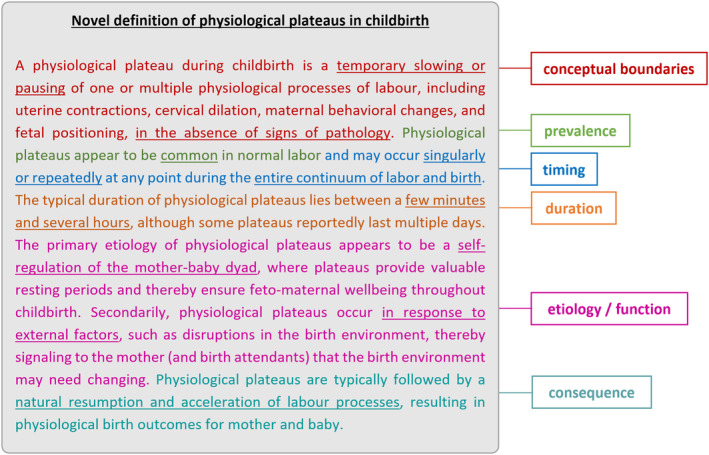
Novel definition of physiological plateaus in childbirth. [Colour figure can be viewed at wileyonlinelibrary.com]

Based on participants' observations and case reports, a comprehensive novel definition of physiological plateaus in childbirth is presented below (Figure 2).

## 4 DISCUSSION

The novel definition of physiological plateaus presented in this manuscript addresses the need for conceptual clarity in this field of research. The term “physiological plateau” can be understood as an umbrella term for any slowing or pausing labor pattern that is considered physiological by the attending midwife and the woman in labor. Since high‐quality research of physiological plateaus remains scarce,^29^, ^31^ this discussion includes references to gray literature.

Midwives in this study described physiological plateaus as a common, normal occurrence during labor, basing this understanding on a cumulative 320 years' experience of caring for women in childbirth. This claim is supported by numerous authors who have researched and/or discussed physiological plateaus in the past decades.^25^, ^33^, ^34^, ^35^, ^36^, ^37^, ^38^, ^39^, ^40^, ^41^, ^42^ The foundation of framing slowing and pausing labor patterns as physiological is the understanding that “there is no justification to implement rigid standardized timelines for physiological labor”.^43^ ^(p.43)^ This understanding recognizes the individuality of each woman and birth and may explain why midwives in this study were not concerned about physiological plateaus in the absence of signs of pathology.

Reliable data on the prevalence of physiological plateaus is currently lacking, and existing reports feature conceptual ambiguity that precludes a meaningful comparison.^29^ For example, one study in the United Kingdom found that uterine contractions plateaued for 52% (*n* = 12) of women on admission to hospital, but the sample size was small and related only to this specific type of plateau.^44^ Midwives in this current study reported that physiological plateaus can occur throughout the entire continuum of labor and birth, which appears corroborated by existing evidence.^29^ Literature also supports the finding that physiological plateaus may last between 10 min and 1 h,^36^, ^37^, ^39^, ^41^, ^42^ up to several hours,^40^, ^45^ or up to several days.^40^, ^46^ Some authors suggest that physiological plateaus may last longer in nulliparous women when compared to multiparous women.^39^, ^42^

Participants in this study theorized that physiological plateaus typically represent a self‐regulation of childbirth, ensuring feto‐maternal well‐being by providing an opportunity for rest, recovery, and adaptation (such as fetal repositioning, and maternal psychological integration). Some midwives from Austria, Ecuador, Germany, and Italy appear to share this view.^40^ Further, several reports support this study's finding that physiological plateaus typically self‐resolve.^34^, ^36^, ^37^, ^40^, ^42^, ^44^, ^45^, ^46^ Given some indication that plateauing labor commonly results in positive birth outcomes for mothers and infants,^24^, ^33^, ^34^, ^35^, ^36^, ^40^, ^45^, ^46^, ^47^ the potential for increasing the rate of physiological birth through better understandings of physiological plateaus could be significant.

Meanwhile, midwives in this study voiced concern that the concept of physiological plateaus does not form “accepted knowledge” in contemporary childbirth discourse, with the exception of early labor and transition (where slowing/pausing labor is somewhat acknowledged as normal).^48^, ^49^ In contrast, when plateaus occur during active first stage or second stage, there is currently no widely acknowledged concept to frame such phenomena as physiological.^32^ Consequently, physiological plateaus occurring during these phases of labor remain at risk of being misinterpreted as labor dystocia.^25^, ^33^, ^44^, ^46^ Different childbirth philosophies among maternity care providers have also been shown to affect how plateauing labor is conceptualized, depending on whether a “dominant medical” or “holistic midwifery” perspective is adopted.^32^ A resulting misinterpretation of physiological plateaus as dystocia appears to contribute to unjustified labor augmentation, and childbirth medicalization.^29^, ^32^

### 4.1 Limitations and recommendations for further research

The self‐selection of participants is likely to have introduced bias as midwives who chose to engage in the interviews evidently had some interest in the subject of slow/pausing labor. While an analysis of respondent demographics and perspectives indicates a heterogeneous sample, those who chose not to join the study may still be midwives who are more inclined to base their judgment strictly on timelines. That is, some midwives may be less likely to consider deviations from what has become an accepted norm as physiologic, either from fear of repercussion or from genuine belief it could be dangerous.

The qualitative design of this research renders quantitative estimates within the findings scientifically unreliable, which affects the prevalence, duration, timing, and consequences of physiological plateaus. Despite some corroborative evidence, these findings require validation through a quantitative study design. The findings on the conceptual boundaries and etiology/function of physiological plateaus are not affected by this limitation. However, it should be explored how women experience physiological plateaus, and which terminology they prefer. To date, the incongruency of extant definitions results in limited data and contributes to a lack of acknowledgement of the existence of physiological plateaus overall.

## 5 CONCLUSION

This research removes conceptual ambiguity by proposing a novel, comprehensive definition of physiological plateaus during childbirth that can be applied in further research. A better understanding and wider recognition of physiological plateaus holds significant potential for a de‐medicalization of childbirth as it can help prevent unjustified labor augmentation. Thereby, research into physiological plateaus addresses priority areas set by the World Health Organization and the International Confederation of Midwives and may help more women and families achieve a healthy, positive birth experience.

## FUNDING INFORMATION

This research was funded through an Australian Government Research Training Program Scholarship as part of a doctoral degree for MW. Additional funding was provided by Edith Cowan University in the form of a Publication Award. The funders were neither involved in the design or conduct of this study nor in the decision to publish this research.

## CONFLICT OF INTEREST STATEMENT

The authors declare no conflicts of interest.

## Data Availability

Anonymized data of this research is openly available via Edith Cowan University's institutional repository Research Online.^50^
